# Usefulness of Xpert MTB/RIF Ultra for rapid diagnosis of extrapulmonary tuberculosis in Tunisia

**DOI:** 10.1038/s41598-024-52130-3

**Published:** 2024-01-26

**Authors:** Imen Bouzouita, Asma Ghariani, Khouloud Ben Dhaou, Sabrine Jemaeil, Leila Essaalah, Sana Bejaoui, Henda Draoui, Naceur El Marzouk, Emna Mehiri, Leila Slim-Saidi

**Affiliations:** 1 National Reference Laboratory for Mycobacteria, LR19SP02, Abderahmane Mami pneumology hospital, Rue de l’hôpital, 2080 Ariana, Tunisia; 2 Faculty of Mathematical, Physical and Natural Sciences of Tunis, University of Tunis El Manar, Campus Universitaire El Manar, 1068 Tunis, Tunisia; 3 Faculty of Pharmacy, University of Monastir, Rue Ibn Sina 5000, Monastir, Tunisia

**Keywords:** Microbiology, Molecular biology

## Abstract

Extrapulmonary tuberculosis (EPTB) remains a challenging diagnosis. The purpose of this study was to assess the accuracy of Xpert MTB/RIF Ultra (Cepheid, USA) for rapid diagnosis of EPTB in Tunisia. Eight hundred and forty-seven extrapulmonary samples collected from 2017 to 2021, were subjected to Xpert MTB/RIF Ultra. Microscopy and culture were performed for all the specimens. The accuracy of Xpert Ultra was evaluated in comparison to the culture. Xpert Ultra diagnosed EPTB with a global sensitivity of 80.66% (74.3–85.75) and specificity of 70.87% (67.31–74.20). The molecular test was most accurate when performed in cerebrospinal fluids, bones and joints and cutaneous specimens showing a sensitivity of 100% and a specificity ranging from 70.60 to 91.11%. In lymph node samples comprising aspirates and biopsies, the sensitivity of Xpert Ultra was high 87.50% (77.23–93.53), however, the specificity was 51.08% (44.67–57.46). For pleural samples, the Xpert Ultra sensitivity was 77.50% (68.34–84.68) ranging from 71.43 to 80% in pleural biopsies and fluids respectively. The specificity in all pleural specimens was 79.56% (74.40–83.91). Xpert Ultra showed promise in the diagnosis of EPTB. The performances varied according to the site of the disease. The test may be more valuable if used in combination with other diagnostic modalities.

## Introduction

Tuberculosis (TB) remains a major health problem worldwide. According to the World Health Organization (WHO), an estimated 10.6 million people had tuberculosis in 2022^1^. In 2019, 7.1 new million cases of TB were notified to the WHO^2^, 16% of which were extrapulmonary (EP) TB cases^2^.

EPTB affects any part of the body other than the lungs with lymph nodes and the pleura being the most frequent localizations^3^. Tunisia, is a middle incidence TB country with a TB incidence estimated at 37 cases/100.000 inhabitants^1^ and a very low prevalence of Human Immunodeficiency Virus (HIV) infection (< 0.1%)^4^. EPTB forms in Tunisia are more common than pulmonary TB and accounted for 62.0% of all TB cases notified in 2022^1^. The prevalence of lymph node TB is high with a steady increase from 2.3/100.000 inhabitants in 1993 to 18.0/100.000 inhabitants in 2017^5^. *Mycobacterium bovis* is the major cause of lymphadenitis TB in the country^5–7^.

The diagnosis of EPTB is challenging. The paucibacillary nature of EPTB and the difficulties in obtaining the samples make the diagnosis by smear staining and culture less sensitive^8^. Since 2010, the WHO has recommended the use of the Xpert MTB/RIF (Cepheid, USA) as initial test for TB and rifampicin (RIF) detection, however, the Xpert was less sensitive in smear negative specimens^9,10^.

To improve the sensitivity, a new version of "Xpert MTB/RIF Ultra" has been developed by Cepheid. Xpert Ultra is a fully automated nested real time PCR that differs from its predecessor in PCR chamber capacity, in the incorporation of two different multicopy targets (*IS*6110 and *IS*1081) and in the optimization of Polymerase Chain Reaction^11^. Xpert Ultra showed an increased sensitivity compared to Xpert MTB/RIF to detect *Mycobacterium tuberculosis* complex (MTBC) in smear negative sputum, HIV + patients, children and extrapulmonary specimens^8,9^. This increased sensitivity is largely due to the “trace call”: a new subcategory result that indicates a detection of minimal bacilli^11^. Since 2017, the WHO has recommended Xpert MTB/RIF Ultra as a substitute to Xpert MTB/RIF^12^.

The aim of this study was to assess the accuracy of Xpert MTB/RIF Ultra in the diagnosis of extrapulmonary TB in Tunisia over a 5-year period, in different site of the disease and specimen nature compared to the culture.

## Material and methods

### Ethical approval

This study is approved by the ethics committee of Abderahman Mami Pneumology Hospital, Ariana, Tunisia.

Due to the retrospective nature of the study, the “ethic committee of Abderahman Mami Pneumology Hospital” waived the need of obtaining informed consent.

All methods were performed in accordance with the relevant guidelines and regulations.

### Samples

From 2017 to 2021, 991 EP samples were tested at the National Reference Laboratory for mycobacteria in Tunisia with Xpert MTB /RIF Ultra (Cepheid, USA).

The culture was the microbiological reference standard (MRS). Information about clinical, histological and radiological TB diagnosis was not available for all the samples. So, a clinical TB diagnosis could not be included as part of a composite reference standard.

### Sample processing

For smear microscopy, all EP liquids were subjected to a cytospin centrifugation before and after decontamination by *N*-acetyl-l-Cysteine NaOH procedure^13^ and a staining with auramine and/or Ziehl Neelsen. Culture was performed on both Lowenstein Jensen and the liquid medium: Mycobacteria Growth Indicator Tube 960 (MGIT 960) (BD, USA).

Biopsy specimens were previously disaggregated and resuspended in 2 ml of saline solution.

Urines and aspirates were previously centrifuged at 3000 rpm for 10 min before decontamination.

### Identification and phenotypic drug susceptibility testing (pDST)

Positive cultures were identified using the SD Bioline Ag MPT64 Kit (Standard Diagnostics, Korea), biochemical tests and the molecular kit: GenoType MTBC (Hain Lifescience, Germany).

The pDST was performed for first line and second line drugs (in case of rifampicin resistance) in MGIT 960 according to the concentrations recommended by the WHO^14–16^.

### Xpert MTB RIF/Ultra assay

The GX Ultra was performed from the pellet. Briefly, 1 ml of the pellet was used and 3 ml of the sample reagent was added. The mixture was well vortexed and kept at room temperature for 15 min. Then, 2 ml were transferred to the cartridge^17^.

### Statistical analysis

Sensitivity, specificity, positive (PPV) and negative predictive values (NPV) were calculated with the open epi software version 3.01 at confidence interval (CI) of 95% and using mycobacterial culture as reference standard .

## Results

### Population and specimens studied

From the 991 EP specimens received during the study period, pediatric (< 15 years) specimens (n = 92), those from patients under antituberculosis treatment (n = 23) and those with contaminated cultures (n = 18) or with invalid or error results (n = 11) were ruled out.

Therefore, a total of 847 EP samples (containing 47 specimens from previously treated patients in the last 5 years) were included: 372 pleural tissues (n = 239) and liquid (n = 133), 295 lymph node biopsies (n = 204) and aspirates (n = 91), 48 Cerebrospinal Fluid (CSF), 34 bones or joints samples (biopsies n = 17 and aspirates n = 17), 21 pericardial aspirates (n = 17) and biopsies (n = 4), 20 peritoneal aspirates (n = 13) and tissues (n = 7), 12 digestive biopsies, 19 cutaneous specimens, 12 genitourinary specimens (urine, sperm, vaginal secretions…), and 14 various EP specimens: breast (n = 5), nasopharyngeal (n = 4), hepatic (n = 3), auricular biopsy (n = 1) and ocular liquid (n = 1) (Supplement 1).

These samples were collected from 760 adult patients (age ≥ 15 years) with a sex ratio of 0.88 and the average age was 46.70 years (15–92 years). All patients were HIV negative except one case.

Microscopy was positive in 78 EP specimens (9.2%), culture was positive in 181 samples (21.36%) and DNA of *M. tuberculosis* complex (MTBC) was detected by means of Xpert Ultra (High, medium, low, very low and trace) in 340 EP samples (40.14%) collected from 309 patients (Table 1).

**Table 1:** Xpert MTB/RIF Ultra results.

Specimens	AFB +	Xpert Ultra +	N° of trace calls	N° of + Xpert from Previously TB treated patients
Lymph node n = 295				
C + 64	21	56	3	7
C− 231	26	113	24	17
Pleural n = 372				
C + 98	14	76	25	5
C− 274	10	56	34	4
CSF n = 48				
C + 3	0	3	0	1
C- 45	1	4	1	0
Bones or joints n = 34				
C + 4	1	4	0	0
C− 30	1	5	4	0
Pericardial n = 21				
C + 0	0	0	0	0
C− 21	0	3	2	0
Peritoneal n = 20				
C + 5	1	4	0	0
C− 15	0	4	4	0
Digestive n = 12				
C + 1	0	0	0	0
C− 11	0	1	1	0
Cutaneous n = 19				
C + 2	1	2	0	0
C− 17	1	5	3	0
Genito-urinary n = 12				
C + 3	1	1	0	0
C− 9	0	3	1	0
Other samples n = 14				
C + 1	0	0	0	0
C− 13	0	0	0	0
Total				
C + 181	39	146	28	13
C- 666	39	194	74	21
**847**	**78**	**340**	**102**	**34**

*AFB +* Acid Fast bacilli staining positive, *C +*  Positive culture, *C−* Negative culture, *CSF* Cerebrospinal Fluids.

The blod values represent the total for each parameter (each column) in the table.

### Xpert MTB/RIF ultra results

The DNA of *M. tuberculosis* complex was detected in 146 out of 181 specimens with a positive MTBC culture (80.66%) and in 194 out of 666 EP specimens with a negative TB culture (29.12%) (Table 1).

Among the 194 specimens, 17 lymph node and 4 pleura specimens came from patients that had a previous TB history (Table1).

The highest rate of positivity by Xpert Ultra in negative culture was found in the lymph node samples (113/231: 48.9%) (Table1). Fifty-six pleura specimens were also found to be positive by Ultra but had negative cultures (56/274: 20.43%) (Table 1).

Trace call results represented 30.0% (n = 102) of all positive Ultra cases. They were found in 38.14% (n = 74) of Ultra positive samples with a negative TB culture (Table 1).

### Xpert MTB/RIF Ultra performances compared to culture and microscopy

Xpert Ultra showed a sensitivity of 80.66% (CI 95% 74.3–85.75) and a specificity of 70.87% (CI 95% 67.31–74.20) to detect *M. tuberculosis complex* DNA in adults presumptive of EPTB compared to culture (Tables 2 and 3), however, the microscopy presented a sensitivity of 21.55% (CI 95% 16.18–28.10) and a specificity of 94.14% (CI 95% 92.10–95.70) compared to culture (Table 3).

**Table 2:** Performances of Xpert Ultra for the diagnosis of extrapulmonary TB compared to culture.

EPTB Form	Total of specimens	Sen %	Spe %	PPV %	NPV %
Lymph nodes	Biopsies (204) Aspirates (91) ALL (295)	88.10 (75–94.81) 86.36 (66.66–95.25) 87.50 (77.23–93.53)	53.09 (45.42–60.61) 46.38 (35.11–58.02) 51.08 (44.67–57.46)	32.74 (24.78–41.84) 33.93 (22.92–47.00) 33.14 (26.48–40.54)	94.51 (87.78–97.63) 91.43 (77.62–97.04) 93.65 (87.97–96.75)
Pleural	Tissues (239) Fluids (133) ALL (372)	71.43 (52.94–84.75) 80 (69.18–87.7) 77.55 (68.34–84.68)	82.00 (76.25–86.60) 71.43 (59.30–81.10) 79.56 (74.40–83.91)	34.48 (23.56–47.33) 75.68 (64.8–84.02) 57.58 (49.05–65.68)	95.58 (91.52–97.74) 76.27 (64.02–85.31) 90.83 (86.51–93.87)
Cerebrospinal Fluid	ALL (48)	100(43.85–100)	91.11(79.27–96.50)	42.86 (15.82–74.95)	100(91.43–100.0)
Bones or joints	Biopsies (17) Aspirates (17) ALL (34)	100 (20.65–100) 100 (43.85–100) 100 (51.01–100)	87.5 (63.98–96.5) 78.57 (52.41–92.43) 83.33 (66.44–92.66)	33.33 (6.15–79.23) 50 (18.76–92.43) 44.44 (18.88–73.34)	100(78,47–100) 100 (74.12–100) 100 (86.68–100)
Pericardial	Biopsies (4) Aspirates (17) ALL (21)	? ? ?	75 (30.06–95.44) 88.24 (65.66–96.71) 85.71 (65.36–95.02)	0 (0–79.35) 0(0–65.76) 0 (0–56.15)	100 (43.85–100) 100 (79.61–100) 100 (82.41–100)
Peritoneal	Biopsies (7) Fluids (13) ALL (20)	75.00 (30.06–95.44) 100.00 (20.65–100) 80.00 (37.55–96.38)	33.33 (6.15–79.23) 83.33 (55.2–95.3) 73.33 (48.05–89.1)	60 (23.07–88.24) 33.33 (6.15–79.23) 50 (21.52–78.48)	50 (9.45–90.55) 100 (72.25–100) 91.67 (64.61–98.51)
Digestive	ALL (12)	0 (0–79.35)	90.91 (62.26–98.38)	0 (0–79.35)	90.91 (62.26–98.38)
Cutaneous	ALL (19)	100 (34.24–100)	70.60 (46.87–86.72)	28.57 (8.22–64.11)	100 (75.75–100)
Genito-urinary	ALL (12)	33.33 (6.14–79.23)	66.67 (35.42–87.94)	25 (4.55–69.94)	75 (40.93–92.85)
Other EP forms	Biopsies (6) Aspirates (8) ALL (14)	? 0 (0–79.35) 0 (0–79.35)	100 (60.97–100) 100 (64.57–100) 100 (77.19–100)	? ? ?	85.71 (48.70–97.43) 87.5 (52.91–97.76) 92.86 (68.53–98.73)
ALL EP samples	847	80.66 (74.3–85.75)	70.87 (67.31–74.20)	42.94 (37.80–48.25)	93.1 (90.55–95.00)

*EP* extrapulmonary, *NPV* negative predictive value, *PPV* positive predictive value, *Sen* sensitivity, *Sp* specificity, *TB* tuberculosis.

**Table 3:** Performances of Microscopy and Xpert Ultra compared to culture.

EP localization		Sensitivity %	Specificity %	PPV%	NPV %
Lymph node N = 295	M Xp	32.81 (22.57–45.00) 87.50 (77.23–93.53)	88.74 (84.02–92.20) 51.08 (44.67–57.46)	44.68 (31.41–58.75) 33.14 (26.48–40.54)	82.66 (77.46–86.87) 93.65 (87.97–96.75)
Pleural N = 372	M Xp	14.29 (8.704–22.56) 77.55 (68.34–84.68)	96.35 (93.41–98.01) 79.56 (74.40–83.91)	58.33 (38.38–75.53) 57.58 (49.05–65.68)	75.86 (71.1–80.06) 90.83 (86.51–93.87)
CSF N = 48	M Xp	0 (0–56.15) 100(43.85–100)	97.78 (88.43–99.61) 91.11(79.27–96.50)	0 (0–79.35) 42.86 (15.82–74.95)	93.62 (82.84–97.81) 100(91.43–100.0)
Bones or joints N = 34	M Xp	25 (4.559–69.94) 100 (51.01–100)	96.67 (83.33–99.41) 83.33 (66.44–92.66)	50 (9.45–90.55) 44.44 (18.88–73.34)	90.63 (75.78–96.76) 100 (86.68–100)
Pericardial N = 21	M Xp	? ?	100 (84.54–100) 85.71 (65.36–95.02)	? 0 (0–56.15)	100 (84.54–100) 100 (82.41–100)
Peritoneal N = 20	M Xp	20 (3.622–62.45) 80.00 (37.55–96.38)	100 (79.61–100) 73.33 (48.05–89.1)	? 50 (21.52–78.48)	91.67(64.61–98.51) 91.67 (64.61–98.51)
Digestive N = 12	M Xp	0 (0–79.35) 0 (0–79.35)	100 (74.12–100) 90.91 (62.26–98.38)	100 (20.65–100) 0 (0–79.35)	78.95 (56.67–91.50) 90.91 (62.26–98.38)
Cutaneous N = 19	M Xp	50 (9.45–90.55) 100 (34.24–100)	94.12 (73.02–98.95) 70.60 (46.87–86.72)	50 (9.45–90.55) 28.57 (8.22–64.11)	94.12 (73.02–98.95) 90.91 (62.26–98.38)
Genito-urinary M N = 12	M Xp	33.33 (6.15–79.23) 33.33 (6.14–79.23)	100(70.08–100) 66.67 (35.42–87.94)	100 (20.65–100) 25 (4.55–69.94)	81.82 (52.3–94.86) 75 (40.93–92.85)
Other * N = 14	M Xp	0 (0–79.35) 0 (0–79.35)	100 (77.19–100) 100 (77.19–100)	? ?	92.86 (68.53–98.73) 92.86 (68.53–98.73)
All EP samples N = 847	M Xp	21.55 (16.18–28.09) 80.66 (74.3–85.75)	94.14 (92.09–95.69) 70.87 (67.31–74.20)	50 (39.17–60.83) 42.94 (37.80–48.25)	81.53 (78.64–84.12) 93.1 (90.55–95.00)

*CSF* cerebrospinal fluid, *M* microscopy, *Xp* Xpert Ultra,*: other extrapulmonary samples.

If EP specimens collected from previously treated patients were ruled out, the test would present a general sensitivity of 79.17% (72.41–84.62), a specificity of 72.63% (69.02–75.96), PPV of 43.46% (38.02–49.07) and NPV of 92.91% (90.31–94.86).

The specificity of Xpert Ultra was low 38.24%” (CI 95% 23.9–54.96) in EP specimens from patients that had a previous TB history during the last 5 years.

All CSF, bones or joints and cutaneous specimens with positive cultures were detected by means of Xpert Ultra showing a sensitivity of 100%, but, their specificity ranged from 70.60 to 91.11% (Table 2).

For lymphadenitis TB, the sensitivity of Xpert Ultra was 87.5% (CI 95% 77.23–93.53) (Tables 2 and 3). Ultra sensitivity in lymph node tissues and aspirates were respectively 88.10% and 86.36%, however, the specificity of the test for lymphadenitis TB was only 51.08% (CI 95% 44.67–57.46).

By excluding lymph node specimens collected from previously treated patients, the test would present a sensitivity of 85.96% (74.68–92.71), a specificity of 54.07% (47.30–60.70), PPV of 33.80% (26.60–41.82) and NPV of 93.40% (87.50–96.61) for diagnosing lymphadenitis TB.

For pleural TB, the microscopy had a sensitivity of 14.29% (CI 95% 8.70–22.56) (Table 3), however, the Xpert Ultra sensitivity was 77.55% (CI 95% 68.34–84.68) with a specificity of 79.56% (CI 95% 74.40–83.91) (Table 2 and 3). The sensitivity of Xpert Ultra in pleural biopsies and liquids was respectively 71.43% and 80.0% (Table 2 and 3).

The lowest sensitivity in EP samples was found in genitourinary samples (33.3%) and digestive specimens (0%) (Tables 2 and 3).

### Xpert MTB/RIF ultra performances in smear negative and positive TB culture samples

Xpert MTB/RIF Ultra showed a sensitivity of 75.35% (CI 95% 67.66–81.71) and a specificity of 70.87% (CI 95% 67.31–74.20) in detecting the DNA of MTBC in samples with smear negative and positive culture (n = 142).

The highest sensitivity in smear negative EP specimens was found in the CSF, bones or joints and cutaneous specimens 100.0% (Table 4).

**Table 4:** Performances of Xpert Ultra in smear negative positive culture specimens.

EPTB form	Total of specimens	Sen %	Spe %	PPV%	NPV%
Lymph nodes	Biopsies (204) Aspirates (81) All (295)	80.77 (62.12–91.50) 82.35 (58.97–93.81) 81.40 (67.38–90.26)	53.10(45.42–60.61) 46.38 (35.11–58.02) 51.08 (44.67–57.46)	21.65 (14.62–30.84) 27.45 (17.11–40.95) 23.65 (17.52–31.11)	94.51 (87.78–97.63) 91.43 (77.62–97.04) 93.65 (87.97–96.75)
Pleural	Tissues (239) Fluids (133) ALL (372)	60.00 (38.66–78.12) 78.13 (66.57–86.5) 73.81 (63.51–82.02)	82.00 (76.25–86.60) 71.43 (59.30–81.10) 79.56 (74.40–83.91)	24.00 (14.30–37.41) 73.53 (62.00–82.55) 52.54 (43.60–61.33)	95.58(91.52–97.74) 76.27 (64.04–85.31) 90.83(86.51–93.87)
Cerebrospinal Fluid	ALL (48)	100(43.85–100)	91.11(79.27–96.50)	42.86 (15.82–74.95)	100(91.43–100.0)
Bones or joints	Biopsies (17) Aspirates (17) ALL (34)	100 (20.65–100) 100 (34.24–100) 100 (43.85–100)	87.5 (63.98–96.5) 78.57 (52.41–92.43) 83.33 (66.44–92.66)	33.33(6.15–79.23) 40.00 (11.76–76.93) 37.5 (13.68–69.43)	100(78.47–100) 100 (74.12–100) 100 (86.68–100)
Pericardial	Biopsies (4) Aspirates (17) ALL (21)	? ? ?	75 (30.06–95.44) 88.24 (65.66–96.71) 85.71 (65.36–95.02)	0 (0–79.35) 0(0–65.76) 0 (0–56.15)	100 (43.85–100) 100 (79.61–100) 100 (82.41–100)
Peritoneal	Biopsies (7) Fluids (13) ALL (20)	66.67 (20.77–93.85) 100 (20.65–100) 75 (30.06–95.44)	33.33 (6.15–79.23) 83.33 (55.2–95.3) 73.33 (48.05–89.1)	50 (15–85) 33.33 (6.14–79.23) 42.86 (15.82–74.95)	50 (9.45–90.55) 100 (72.25–100) 91.67 (64.61–98.51)
Digestive	ALL (12)	0 (0–79.35)	90.91 (62.26–98.38)	0 (0–79.35)	90.91 (62.26–98.38)
Cutaneous	ALL (19)	100 (20.65–100)	70.60 (46.87–86.72)	16.67 (3.00–56.35)	100 (75.75–100)
Genito-urinary	ALL (12)	0 (0–65.76)	66.67 (35.42–87.94)	0 (0–56.15)	75 (40.93–92.85)
Other EP forms	Biopsies (6) Aspirates (8) ALL (14)	? 0 (0–79.35) 0 (0–79.35)	100 (60.97–100) 100 (64.57–100) 100 (77.19–100)	? ? ?	85.71 (48.69–97.43) 87.5 (52.91–97.76) 92.86 (68.53–98.73)
ALL EP samples	847	75.35 (67.66–81.71)	70.87 ( 67.31–74.20)	35.55 (30.35–41.11)	93.1 (90.55–95.00)

EP: extrapulmonary, NPV: negative predictive value , PPV: positive predictive value, Sen: sensitivity, Sp: specificity , TB: tuberculosis.

The sensitivity in smear negative lymph nodes was important “81.40%” (CI 95% 67.38–90.26). In fact, 35 out of 43 smear negative lymph node samples were detected by Xpert Ultra (Supplement 1).

Xpert Ultra was positive in 73.81% of pleura specimens that presented a smear negative microscopy and a positive TB culture (62 out of 84) (Supplement 1). The Xpert MTB/RIF Ultra sensitivity in the pleural liquid and biopsies was respectively 73.81% (CI 95% 63.51–82.02) and 60.00% (CI 95% 38.66–78.12%) (Table 4).

### Xpert MTB/RIF Ultra and rifampicin detection in EP specimens

The DNA of MTBC was detected in 340 EP specimens (340/847: 40.14%) collected from 309 patients (309/760: 40.65%). One hundred and thirty-five of the patients had a positive TB culture (135/309: 43.68%).

**Figure 1:**
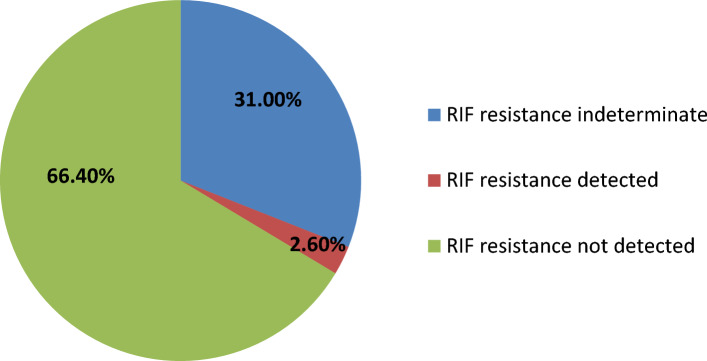
Rifampicin detection results by Xpert MTB/RIF Ultra.

Rifampicin resistance detection results are summarized in Fig. 1.

The prevalence of rifampicin resistance in EPTB patients with Xpert Ultra was 2.6%. In fact, 8 patients showed a resistance to RIF: 7 cases had a pleural TB and one patient had a lymphadenitis TB. This resistance was confirmed only in 3 patients with a positive culture.

Ninety-five patients had a DNA trace and indeterminate RIF resistance results (30.7%), 28 of them had a positive culture (29.4%) and the pDST showed a sensitivity to RIF.

### Molecular identification of positive extrapulmonary tuberculosis cultures

In our study, 181 EP samples had a positive cultures coming from 165 adult patients. The molecular identification of EPTB cultures using the GenoType MTBC showed that *M. tuberculosis* was involved in 148 EP cases (148/165. 89.7% of patients), however *M. bovis* was found in 17 patients (17/165, 10.3% of patients) (Supplementary SI).

We found that *Mycobacterium bovis* was responsible for 22.4% of lymphadenitis TB cases (13/58). Identification results were reported in the supplementary table “SI”.

## Discussions

Diagnosis of EPTB is challenging. In fact, the clinical symptoms and the radiological features are not specific. The histological examination is not widely available and it has shown a reduced specificity for EPTB diagnosis^18^. In addition, specimen collection from relatively inaccessible sites is difficult and the paucibacillary nature of EPTB is the main difficulty facing the bacteriological diagnosis^18,19^.

The molecular methods are useful tools to improve the accuracy of the diagnosis of EPTB^8^. Xpert MTB/RIF Ultra has shown a high sensitivity and specificity to detect pulmonary TB in adults, children and HIV + patients^10,20,21^, however, so far few studies have been carried out to evaluate its performance for the diagnosis of EPTB.

In the present study, the accuracy of Xpert Ultra was evaluated using a large collection of specimens obtained from different localizations (n = 847). The pleura (n = 372) and lymph node (n = 295) sites were the most common sites of the disease.

In comparison with the microbiological reference standard, Xpert Ultra showed a sensitivity of 80.66% (95% CI 74.3–85.75) in diagnosing EPTB. This sensitivity is higher than the one reported by Sekyere et al. in a high endemic TB setting in South Africa^22^: 69.23%, but lower than the sensitivity reported in Italy: a low prevalence TB setting”: 95.6% (95% CI 84.8—99.5)^9^.

Xpert Ultra sensitivity was high in smear negative positive culture TB cases (n = 142): 75.35% (67.66–81.71). This finding is similar to the one reported in the study of Perez-Risco et al. 75.9% (95% CI 66.6–83.4%) which evaluated 108 smear negative extra pulmonary specimens and TB culture positive^20^.

Xpert Ultra specificity in this study was low 70.87% (67.31–74.20**)** compared to the specificity found in low TB prevalence areas^9,23^. Several reasons could explain this findings: first, it is known that culture is an imperfect reference standard in EPTB paucibacillary cases^18^, second, inefficient specimen collection, or worst sampling^8^ could lead to a false negative culture. Finally, the new category “trace call” represents 38.14% (n = 74/194) of all the Xpert positive-cases found in negative TB cultures decreasing the specificity of the test to diagnose EPTB.

According to the WHO, “trace call” results should be considered as true positive for persons living with HIV, children, and for extrapulmonary specimens^11,24^.

For HIV-negative persons, trace call was considered positive in those without a prior or a recent history of TB^25^. Therefore, positive Xpert Ultra result, including trace calls, should be interpreted carefully in patients with a previous TB history. This also explains the low specificity found for specimens collected from previously treated patients (n = 47) in this study 38.24%” (CI 95% 23.9–54.96). For this category of patients, Xpert Ultra is not useful for TB diagnosis and only culture could help to take an accurate decision. Nevertheless, the number of specimens collected from patients with prior TB history is low (n = 47) and ruling out these samples did not enhance notably the specificity of the test.

Tuberculous meningitis has a high morbidity and mortality. The rapid diagnosis is a priority. The WHO recommends the use of Xpert MTB/RIF and Xpert MTB/RIF Ultra as initial rapid test for diagnosing this TB form^26^.

In our study, Xpert Ultra sensitivity and specificity were high in CSF: 100.00% and 91.11% respectively. Kohli and his colleagues reported*,* a pooled sensitivity and specificity of Xpert Ultra of 89.4% (79.1–95.6%) and 91.2% (83.2–95.7) compared to culture^8^, however, the pooled sensitivity and specificity for Xpert MTB/RIF were 71.1% and 96.9%^8^.

Lymphadenitis TB is the most common EPTB form in Tunisia and *M. bovis* is the most frequently involved in lymph node TB cases^5–7^. Xpert Ultra showed a high and similar sensitivity in both tissues “88.1”% and aspirates “86.3%” compared to culture. Thus,

lymph node aspirates could be used as initial test when lymphadenitis TB is suspected, as has been suggested by Antel et al.^27^. In fact, this kind of specimens is easy to perform and does not require any special equipment.

Xpert Ultra sensitivity in our study was lower than Xpert MTB/RIF sensitivity reported in Tunisia by Ghariani et al. (n = 174 lymph node samples): 87.5% versus 94.9%^6^. Nevertheless, Xpert Ultra specificity was high compared to the one reported by Ghariani et al. (51.08% vs. 37.9%)^6^. The low specificity of the molecular tools compared to culture could be linked to a poor sample handling and a harsh decontamination of the samples with *N*-acetyl-l-cysteine sodium hydroxide which could distort the culture results.

It was reported that *M. bovis* could be responsible for 78% of lymphadenitis TB cases in Tunisia based on a previous national investigation established during the period 2012–2014^5^.

In the present study, *M. bovis* was only involved in 22.4% of lymph nodes TB cases during the study period”. This finding could be explained by the fact that the samples received in the present study came mainly from Tunis capital region whereas the national investigation covered all the regions of the country including those where *M. bovis* predominates such as southern Tunisia.

Pleural tuberculosis is the most common localization of EPTB worldwide. Compared to Xpert MTB/RIF, the sensitivity of Xpert Ultra in pleural fluids and pleural biopsies was improved and it represented respectively 80.0% (69.18–87.7) and 71.43% (52.94–84.75).

The pooled sensitivity of Xpert MTB/RIF, according to Cochrane’s review was 50.9% (39.7–62.8) in pleural fluids and only 30.5% (3.5–77.8%) in pleural biopsies^18^. This enhanced sensitivity in pleural fluids with Xpert Ultra suggests that pleural liquids could be used as initial diagnostic test for presumptive pleural TB cases as proposed in the meta analysis of Aggarwal et al.^28^. Xpert Ultra sensitivity was also improved in peritoneal fluids: 100% (20.65–100) versus 59% for Xpert MTB/RIF^26^. Unlike to peritoneal fluids, the performance of Xpert Ultra seems limited in the remaining digestive specimens: intestinal, celiac and gastric (sensitivity of 0%, specificity of 90.91%).

Osteoarticular and skin TB are uncommon TB forms. Xpert Ultra showed a very high performance in diagnosing these forms in comparison to the reference standard: 100% of sensitivity (specificity was respectively: 83.33% and 70.60%). A good performance of Xpert Ultra for the diagnosis of TB in bones and joints has been previously reported by Sun et al. who found a sensitivity of 96% (87–100%) and a specificity of 97% (85–100%)^29^.

Regarding genitourinary TB, the test detected only one positive sample out of 3 positive TB samples. Further researches are needed to better evaluate the assay in rare EPTB localizations.

“Trace call and RIF resistance indeterminate result” were found in 95 patients and RIF resistance information was provided for 29.4% of these patients by culture. The lack of RIF susceptibility result could be problematic in high MDR incidence area. In fact, MDR-TB cases could be missed requiring culture to perform a phenotypic DST, which delays the prescription of an adequate treatment.

The prevalence of RIF resistance in EP samples according to Xpert Ultra was low in this study (n = 8 patients, 2.6%) as Tunisia is a low Rifampicin Resistance -MDR TB incidence country^1,30^.

The absence of a composite reference standard represents a major limitation of this study. In fact, including clinical signs, radiological and histological findings could affect the sensitivity and the specificity of Xpert MTB/RIF Ultra by increasing or decreasing the values reported in this research. In addition, the size of the samples for some EP localizations such as pericardial, urogenital, digestive and cutaneous was small and may not reflect the real performances of the test.

## Conclusions

In conclusion, a good performance of Xpert MTB/RIF Ultra was observed in this study proving its efficiency in diagnosing extrapulmonary tuberculosis in Tunisia especially CSF, bones and joints, cutaneous, lymph nodes and pleural samples. The enhanced sensitivity of the test in lymph node aspirates and pleural fluids suggests that they could be used as initial test to diagnose lymphadenitis and pleural TB respectively.

The generally reduced specificity observed indicates that the test may be more valuable if used in combination with clinical, radiological and histological results.

Given the poor performance of microscopy as a rapid diagnostic method, Xpert MTB/RIF Ultra must be adopted as initial diagnostic test in the event of any suspicion of extrapulmonary tuberculosis.

### Supplementary Information

Supplementary Information 1.

Supplementary Information 2.

## Data Availability

The datasets generated and analysed during the current study are available in the supplementary file “SI”. All data generated or analysed during this study are included in this published article and its supplementary information files.
